# Navigating COVID-19 and Long-Term Care: Dementia Caregivers' Challenges and Strengths

**DOI:** 10.1093/geroni/igab046.033

**Published:** 2021-12-17

**Authors:** Lauren Mitchell, Elizabeth Albers, Robyn Birkeland, Henry Stabler, Jinhee Cha, Joseph Gaugler

**Affiliations:** 1 Emmanuel College, Boston, Massachusetts, United States; 2 University of Minnesota, Minneapolis, Minnesota, United States; 3 University of Minnesota, University of Minnesota, Minnesota, United States

## Abstract

Persons with dementia living in long-term care settings have been especially affected by the COVID-19 pandemic, and their family caregivers have had to cope with numerous additional stressors during this time. We conducted 20 semi-structured interviews and gathered open-ended survey data from N=104 caregivers participating in an ongoing intervention trial at the start of the COVID-19 pandemic. Open-ended questions explored the difficulties caregivers have experienced in caring for and supporting a relative in long-term residential care. Caregivers provided their perspectives about services and supports that have facilitated coping with uncertainty, anxiety, and loss during the pandemic, and identified resources and strengths they have found helpful in caring for their relatives. Thematic analysis was used to identify themes reflecting the key challenges and supports that have emerged for caregivers, and to highlight caregivers' recommendations for promoting their and their relatives' well-being during this crisis.

